# HEALTH, WORRY, AND FOOD INSECURITY IN LOW-INCOME U.S. ADULTS: AN EXPLORATION OF MIDLIFE VULNERABILITY

**DOI:** 10.1093/geroni/igz038.1835

**Published:** 2019-11-08

**Authors:** Lisa Miller

**Affiliations:** University of California, Davis, Davis, California, United States

## Abstract

Food insecurity, defined as the inability to afford and access nutritious foods to eat, is associated with poor health, higher healthcare costs, and increased risk of mortality (Gundersen et al., 2018). Moreover, food insecurity appears to accelerate aging processes. For example, food insecurity is associated with inadequate nutrition, which expedites the loss of muscle mass, increases mobility problems, increases financial worry, and increases the risk of frailty. While a good deal is known about food insecurity in later life, far less is known about midlife, which may be a time of unexplored vulnerability. We examined food insecurity in a sample (n=17,866; 2014 NHIS) of low-income (PIR<3) young, early-middle, late-middle, and older adults (18-84), focusing on health challenges (chronic conditions, functional limitations) and financial worry as predictors and whether their effects varied with age. Multinomial logistic regression was used to assess the association of predictors with food insecurity and determine whether associations differed by age group, adjusted for covariates (e.g., sex, race/ethnicity, education, social security). Food insecurity rates were highest in late- (37.5%) and early- (36.0%) midlife followed by young (33.7%) and older (20.2%) adults. Age moderated the relationship between food insecurity and risk factors (p < .05 for both) such that health was stronger- but financial worry was weaker- in midlife (due to higher food insecurity in low- and high- worry groups). Midlife is a period of increased vulnerability to food insecurity, particularly for those with health challenges. Research is needed to inform prevention strategies to help ensure optimal aging.

